# Research progress on the uptake and transport of antimony and arsenic in the soil-crop system

**DOI:** 10.3389/fpls.2025.1610041

**Published:** 2025-08-21

**Authors:** Jianyang He, Ke Yang, Sheng Wang, Yingmei Li, Li Bao, Naiming Zhang

**Affiliations:** ^1^ College of Resources and Environment, Yunnan Agricultural University, Kunming, Yunnan, China; ^2^ Yunnan Engineering Research Center for Soil Fertilization and Pollution Remediation, Kunming, China; ^3^ College of Resources, Environment and Chemistry, Chuxiong Normal University, Chuxiong, Yunnan, China

**Keywords:** antimony, arsenic, co-contamination, uptake and translocation, health risk

## Abstract

Antimony (Sb) and arsenic (As) are homologous elements that pose significant threats to the ecological security of soil-crop systems and the health of agricultural products due to their co-contamination. Although they share similarities in plant uptake and translocation, significant knowledge gaps remain regarding the uptake mechanisms of Sb, especially Sb(V), and its interactions with As. This review systematically summarizes the sources, chemical speciation, and bioavailability-regulating factors (e.g., pH, redox conditions, microbial communities) of Sb and As in soil-crop systems, focusing on their uptake pathways, translocation characteristics, and synergistic or antagonistic effects under co-contamination. Comparative analyses suggest that As(V) is taken up through phosphate transporters, whereas the transport mechanism of Sb(V) remains unclear. Under co-contamination, As may enhance Sb accumulation by altering membrane permeability; however, differences in their translocation efficiency and speciation transformation lead to antagonistic effects. Additionally, soil physicochemical properties and plant species significantly influence Sb-As toxicity responses. The detoxification mechanisms of hyperaccumulators (e.g., *Pteris vittata*) offer novel insights for remediation technologies. By integrating multidisciplinary findings, this review identifies key challenges in co-contamination research and provides theoretical foundations for farmland remediation and risk management based on bioavailability regulation.

## 1 Introduction

Soil contamination is a global issue, with varying degrees and types depending on contamination sources, among which heavy metal contamination is especially prominent (Sun et al., 2023; Xie et al., 2016). Many heavy metals in soil, including nickel (Ni), cobalt (Co), zinc (Zn), chromium (Cr), manganese (Mn), mercury (Hg), copper (Cu), and cadmium (Cd), have been listed as high-risk pollutants by the U.S. Agency for Toxic Substances and Disease Registry (ATSDR) and the U.S. Environmental Protection Agency (EPA). These elements are also present in industrial wastewater, posing a significant threat to ecosystems (Azhar et al., 2022). In China, according to the “National Soil Pollution Survey Bulletin,” arsenic (As) ranks as the third most polluted element, following cadmium (Cd) and nickel (Ni). Antimony (Sb), once overlooked as a potentially toxic element, has become a global concern due to its widespread use in industry and daily life. Excessive accumulation of potentially toxic elements can damage soil health, hinder plant growth, and disrupt microbial activity, severely affecting the structure and function of ecosystems (Chen et al., 2023; Zhang et al., 2021). Reports indicate that global antimony mining reached 110,000 tons in 2022, with China being the largest producer, accounting for 55% of global output (Wang et al., 2023a). Antimony ore mining is often accompanied by the generation of arsenic, meaning that mining and smelting processes typically lead to co-contamination by Sb and As. Given their similar chemical properties and toxicity, these two elements are prioritized as pollutants (Pierart et al., 2015; Tan et al., 2018).

Sb and As primarily enter the human body through the food chain, posing a threat to human health, with rice and vegetables as the main sources of exposure. Previous studies have shown that leafy vegetables are the second-largest source of Sb exposure, accounting for 26% of total Sb intake (Feng et al., 2013a; Guo et al., 2021). Health risk assessments reveal that the hazard quotient (HQ) of Sb in vegetables exceeds the safety limits recommended by the World Health Organization (WHO). Surveys of lifestyle habits among residents near antimony mining areas show that Sb is a major health risk, with the average daily intake significantly exceeding the tolerable intake (Wu et al., 2011). Globally, leafy vegetables are one of the main pathways for heavy metal exposure, particularly in Asian countries. Therefore, understanding the uptake and transport mechanisms of Sb and As in the soil-crop system is essential for protecting the ecological environment and human health.

Sb and As, as elements of the same group, exhibit similar redox characteristics in the environment, leading to potential commonality in their environmental mobility and bioavailability. This geochemical similarity has led researchers to often infer the ecological toxicity effects of Sb based on As, but it is important to emphasize that the specific uptake mechanisms of Sb remain significantly underexplored (Wilson et al., 2010). Current studies confirm that As(III) and Sb(III) share water channel protein transport systems, while the phosphate co-transport mechanism for As(V) is well understood. However, the uptake pathway of Sb(V) remains unclear, and this knowledge gap limits our understanding of its environmental behavior (Feng et al., 2019). It is noteworthy that different plant groups exhibit significant differentiation in their preference for Sb uptake, such as the root enrichment characteristics of hyperaccumulators like *Pteris vittata* and *P. cretica* for Sb(III) (Tisarum et al., 2014). This suggests that the uptake and transport mechanisms of Sb in plants may be more diverse than previously expected, and this diversity is useful for developing targeted plant remediation strategies. Of greater concern is that the synergistic toxic effects of Sb-As co-contamination significantly increase the risk of accumulation of both pollutants in crops (Egodawatta et al., 2020). These findings suggest that considering Sb and As as associated pollutants in integrated ecological risk assessments should be an important focus for future contamination control research.

This review summarizes the sources of Sb and As in soil and their health hazards to ecological systems, focusing on the uptake and transport mechanisms of Sb and As in crops, especially under co-contamination conditions, and their impact on plant growth and heavy metal accumulation. Although previous studies have revealed the uptake pathways and similarities of Sb and As, the uptake mechanism of Sb(V) remains unclear, and the long-term effects of synergistic toxicity under co-contamination remain underexplored. Future research should focus on revealing the interactions between Sb and As, particularly the uptake pathways of Sb, and their migration and transformation in the soil-crop system. This will help to assess the potential risks they pose to agricultural product safety and public health more comprehensively and provide a scientific basis for ecological environment management.

## 2 Sources of Sb and As

**Figure 1:**
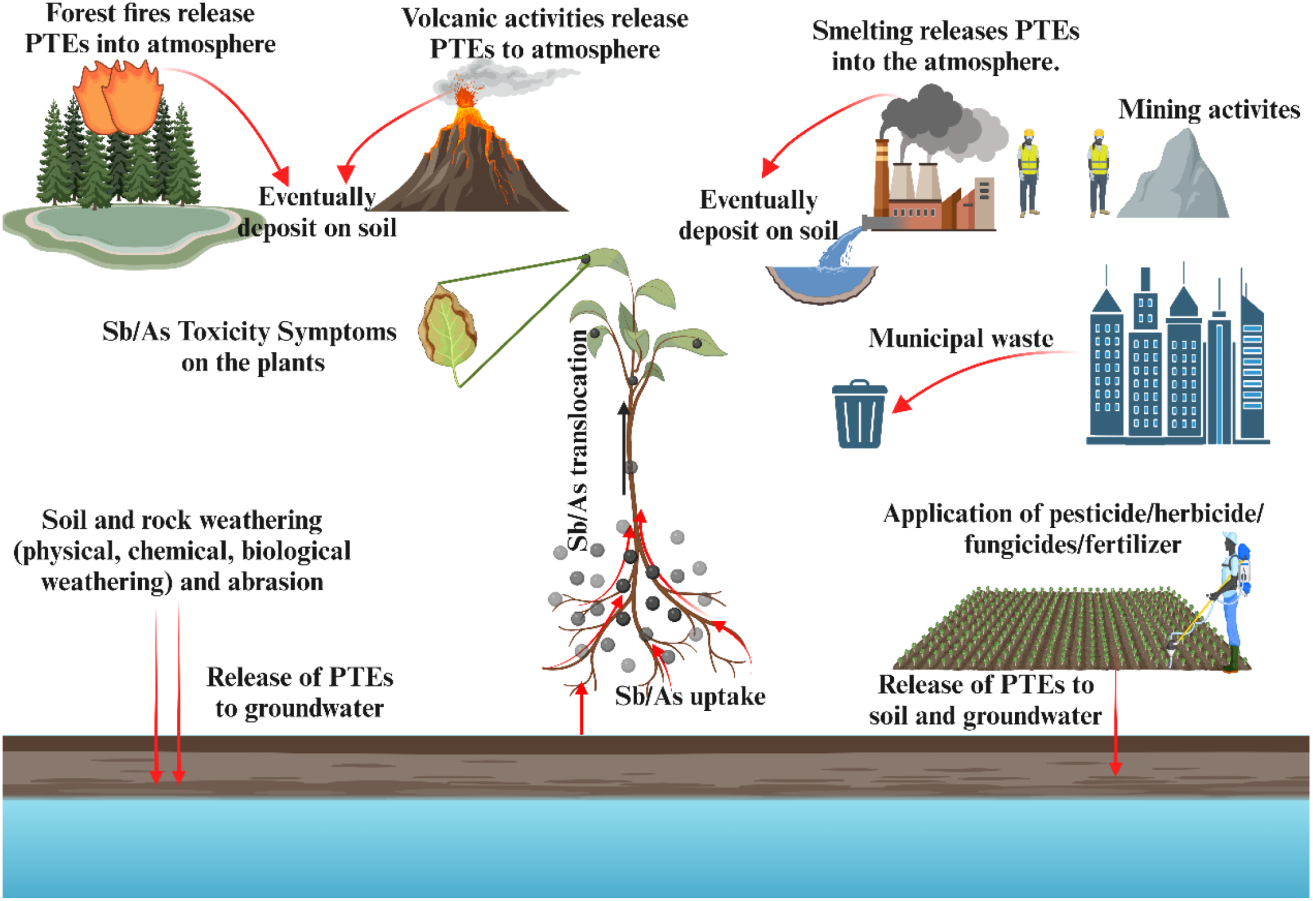
The sources of Sb and As pollution. In the environment, including both natural sources (e.g., weathering, volcanic activity) and anthropogenic sources (e.g., mining, industrial processes, agricultural activities). Arrows indicate major pathways of release and transfer between environmental compartments.

Sb and As share similar geochemical properties and toxicity due to their identical s²p³ electronic configurations, which result in oxidation states of -III, 0, +III, and +V, respectively, in their various chemical forms. Both elements exist in organic and inorganic forms in the environment, and mining activities and compound utilization increase their concentrations. Therefore, human activities have a more significant environmental impact than natural sources (Bolan et al., 2022) (**Figure 1**).

### 2.1 Natural sources

Sb is commonly found in various environmental matrices. Although Sb concentrations in the Earth’s crust are minimal (0.2–0.3 μg·g^-^¹), these levels vary significantly in rocks (0.2–300 μg·g^-^¹) (Haider et al., 2024). Its inorganic forms include antimony trioxide (Sb_23_), antimony pentoxide (Sb_25_), antimony trisulfide (Sb_23_), and other sulfide minerals (Vikent’Eva and Vikentev, 2016). Natural processes such as weathering, volcanic eruptions, wind dust, and forest fires release Sb into the environment, with about 5% of global Sb emissions originating from volcanic eruptions (Quiroz et al., 2016; Warnken et al., 2017). The concentration of Sb in sedimentary rocks, soil, and water is 0.15–2 mg·kg^-^¹, 0.3–8.6 mg·kg^-^¹, and < 1 μg·g^-^¹, respectively (Pierart et al., 2015), with concentrations influenced by the parent material, typically ranging from 0.2–10 mg·kg^-^¹ and usually below 1 mg·kg^-^¹ (Tschan et al., 2009). The natural sources of As are similar to those of Sb and are also affected by volcanic eruptions, biological volatilization, and soil erosion processes (Meharg and Meharg, 2021).

### 2.2 Anthropogenic sources

Industrialization has significantly increased Sb and As contamination, especially in mineral-rich regions, where erosion may elevate background levels (Wen et al., 2020; Zhang et al., 2018). The mining and smelting of sulfur-containing minerals, along with chemical waste (such as arsenic alkaline residues and desulfurization residues), release Sb and As into the environment through weathering and rainfall (Bolan et al., 2022; Li et al., 2024; Zhuang et al., 2018). Globally, regions such as northern Vietnam, Portugal, and China have higher concentrations of Sb in soil due to mining and industrial emissions. For example, antimony mines in northern Vietnam and Portugal have concentrations of 15699 mg·kg^-^¹ and 5956 mg·kg^-^¹, respectively, while in China, antimony contamination is particularly severe in areas like the Xishan tin mine (5045 mg·kg^-^¹) and the abandoned arsenic processing site in Hechi (2420 ± 217 mg·kg^-^¹ for Sb, 6547 ± 362 mg·kg^-^¹ for As) (Bolan et al., 2022; Cappuyns et al., 2021; Hu et al., 2017; Pratas et al., 2005; Zhou et al., 2022). Additionally, sewage sludge, motor vehicle emissions, industrial waste leakage, and plastic leachate are also sources of Sb and As contamination (Bolan et al., 2022). Furthermore, shooting ranges using lead bullets containing 2-8% Sb also contribute to soil contamination with Sb, Pb, and Cu. Switzerland emits approximately 25 tons of Sb annually, while the U.S. emits up to 1900 tons of Sb annually (Mariussen et al., 2017; Sanderson et al., 2015; Wan et al., 2013).

## 3 Uptake and transport of Sb and As in the soil-plant system

In the soil-plant system, the uptake and transport of Sb and As are influenced by a combination of soil conditions and plant metabolism, with bioavailability directly affecting their uptake and transport within plants. The uptake efficiency of both elements in plants is influenced by soil pH, organic matter, and redox potential. As is typically taken up by plants in the form of arsenate [As(V)], competing with phosphate, while arsenite [As(III)] is more bioavailable due to its higher solubility and enters the plant through aquaporins. Similarly, Sb uptake depends on its chemical form; Sb(III) is more bioavailable under reducing conditions and may be taken up through phosphate or sulfate transporters. The higher the bioavailability, the greater the plant’s ability to take up these elements, which in turn affects their accumulation and toxicity within the plant.

### 3.1 Bioavailability of Sb and As

Traditional ecological risk assessments are typically based on the total concentration of heavy metals, but this indicator fails to accurately reflect geographical variations and biological toxicity (Zhang et al., 2024). The toxicity of heavy metals depends on their bioavailability, which is influenced by their oxidation state and chemical form (Caporale and Violante, 2016). The uptake of trace elements by plants is a crucial step for their entry into the food chain, relying on the migration of these elements from the soil to the root and their passage through root cell membranes, subsequently being transported to the xylem and storage sites such as stems, leaves, seeds, and fruits (John and Leventhal, 1995). The transfer of elements from the soil to the root is a key limiting factor for plant uptake, and this process is influenced by the concentration of elements in the soil pore water, as well as local physicochemical conditions such as moisture, pH, and redox potential. A small amount of heavy metals in the soil exists in free or complexed forms in the pore water, available for plant uptake, while reaching a dynamic equilibrium with the metals in the solid phase (Antoniadis et al., 2017a). This equilibrium is affected by factors such as pH, humidity, organic carbon content, redox conditions, and the levels of carbonates and sulfides, all of which can be altered by anthropogenic contamination (Kim et al., 2015; O’Connor et al., 2019). Soil pH is a key environmental factor that controls the bioavailability of heavy metals and significantly influences their solubility (Antoniadis et al., 2017b). For example, Sb is more readily taken up by plants in alkaline soils (pH 8.39) compared to acidic soils (pH 4.91) (Zhong et al., 2020). Furthermore, as soil redox potential (Eh) decreases, the bioavailability of Sb increases (Zhu et al., 2020). Soil microorganisms, such as bacteria, archaea, and fungi, can also regulate Sb bioavailability by altering its chemical form (Long et al., 2020). Uptake of Sb by plants often leads to significant accumulation in the plant, which can adversely affect plant health (Maresca et al., 2020). Moreover, plant species and the relative abundance of essential nutrients can also influence metal uptake. Bioavailable essential nutrients (such as Ca, Mg and Fe) can reduce the uptake of non-essential metals, and interactions between multiple elements may also affect metal bioavailability (Van Caneghem et al., 2016; Yuan et al., 2023). For instance, in the pakchoi (*Brassica rapa subsp. chinensis*)-soil system, an increase in iron oxide concentration can reduce the bioavailability of Sb and lower the accumulation of Sb in pakchoi. The core mechanism involves surface adsorption, coprecipitation, and redox reactions that immobilize Sb(V) as stable Fe-Sb oxide complexes or oxidize Sb(III) into the less mobile Sb(V), thereby reducing plant uptake of Sb (Chang et al., 2022). It is noteworthy that soils in antimony mining areas are often contaminated not only with Sb but also with As, and the factors influencing the bioavailability of Sb also affect that of As.

### 3.2 Sb uptake and transport mechanisms

Building upon the identified natural and anthropogenic sources of Sb and As (Section 2) and their bioavailability in soil-plant systems (Section 3.1), the internalization and translocation of these metalloids are critically governed by plant-specific mechanisms. The following sections elucidate the physiological and molecular basis of Sb uptake and transport within crops.

#### 3.2.1 Mechanisms of Sb uptake

##### 3.2.1.1 Predominant Sb species and uptake pathways

In Sb-contaminated environments, Sb in plant tissues primarily exists as Sb(V), comprising about 95% of the total Sb content (Wu et al., 2011). Despite limited research on Sb uptake mechanisms, it is widely accepted that Sb is mainly absorbed through apoplastic and symplastic pathways, with transporter proteins potentially involved in the latter. The apoplastic pathway transports water and solutes through cell walls and intercellular spaces, whereas the symplastic pathway moves them through the cytoplasm of interconnected cells via plasmodesmata (Feng et al., 2013a; Tschan et al., 2009).

**Figure 2:**
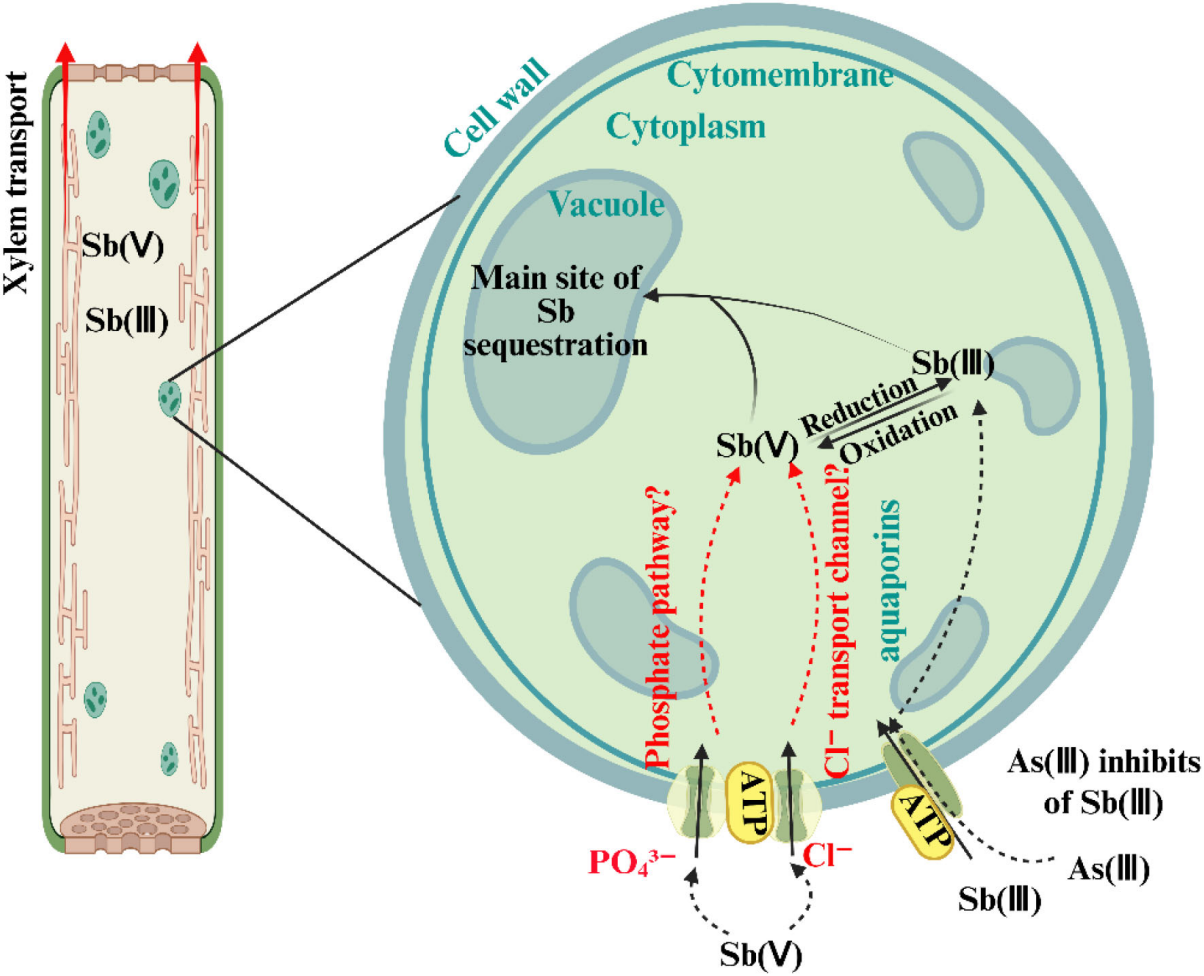
Schematic diagram illustrating the uptake pathways of Sb(III) and Sb(V) in plants, including the transport from roots to stems, transformation between Sb species, and differences in sequestration at the subcellular level (e.g., vacuolar compartmentalization). The diagram also highlights the involvement of transport proteins such as aquaporins [for Sb(III)] and phosphate transporters [for Sb(V)]. Adapted from Zhu et al. (2020).

Tschan et al. (2009) proposed detailed mechanisms for Sb(V) uptake via these pathways (**Figure 2**). The symplastic pathway involves selective intercellular transport. When Sb moves towards the root stele via the apoplast, the Casparian strip serves as a barrier, redirecting solutes into the symplast through endodermal cells. At this point, solutes must cross cell membranes into the symplastic stream, a process potentially mediated by specific transporters. The Pht1;4 gene, recently identified in *Arabidopsis thaliana*, plays a critical role in Sb(V) transmembrane transport. Knockout of Pht1;4 (mutant line M-P4) significantly reduced Sb(V) uptake in roots, resulting in 25–50% lower Sb accumulation compared to wild-type plants under 10 mg·L^-^¹ Sb(V) exposure (Dong et al., 2025). In contrast, the apoplastic pathway depends on diffusion through cell wall pores and intercellular spaces. Although the root cortex apoplast directly interfaces with external solutes, the Casparian strip typically redirects their movement into the symplast. However, at sites such as lateral root junctions or root tips, where the Casparian strip is immature or damaged, Sb(V) may bypass cell membranes and move directly from roots to shoots via apoplastic “shortcuts”. This phenomenon is similar to the movement of larger molecules such as ethylenediamine-N,N′ -disuccinic acid (EDDS) (Jung et al., 2002). These dual mechanisms suggest that Sb(V) uptake and transport in plants involve both apoplastic diffusion and symplastic transport proteins. Nevertheless, the subcellular distribution, chemical speciation, and physiological effects of Sb in plants remain poorly understood and require further investigation.

##### 3.2.1.2 Species-specific uptake preferences

Different crops—and even different tissues within the same crop—can exhibit varying uptake efficiencies for different oxidation states of Sb. For instance, rice roots show a higher affinity for Sb(III) than Sb(V) (Ren et al., 2014), whereas cabbage stems and leaves accumulate substantially more Sb(V) than Sb(III) (Wu et al., 2020), suggesting that leafy vegetables may preferentially transport Sb(V) via the xylem. Additionally, Sb uptake may occur through the plant’s uptake system for essential elements (Tschan et al., 2009). Studies indicate that As(III) can inhibit Sb(III) uptake (Meharg and Jardine, 2003). When As(III) is added alongside Sb(III) and Sb(V), it suppresses Sb(III) uptake but does not affect Sb(V) uptake, suggesting that Sb(III) and Sb(V) follow distinct uptake pathways (Brochu et al., 2003). Sb(III) uptake appears to share mechanisms with As(III), whereas Sb(V) uptake is unaffected by As(III). As an analogue of arsenate [As(V)], AsO_4_³^-^ is taken up via pathways similar to those of PO_4_³^-^ [P(V)], whereas Sb(OH)_6_^-^ follows a different route, likely due to differences in crystal structure. These structural differences lead to distinct uptake and metabolic mechanisms for Sb(V) and As(V) in plants (Tschan et al., 2008).

##### 3.2.1.3 Dose effects and competitive interactions

Sb uptake exhibits clear dose-dependency, which is further complicated by the presence of competitive inhibitors. Sb(V) uptake is influenced by multiple factors, particularly in the presence of competing ions, resulting in a dose-dependent uptake efficiency. For example, high levels of PO_4_³^-^ significantly enhance As uptake in some plants, but its effect on Sb uptake varies (Chen et al., 2002; Tu and Ma, 2003). Similarly, Sb(V) and As(V) exhibit dose-dependent antagonism: high Sb(V) levels inhibit As(V) uptake, and vice versa (Müller et al., 2013; Wan et al., 2016). This competitive suggests that Sb(V) uptake is modulated by interactions with other mineral elements within the plant. Further studies have shown that phosphate (P(V)) concentrations significantly affect Sb uptake. For example, high P(V) levels slightly inhibit Sb(V) uptake in rice (Ren et al., 2014) strongly inhibit it in wheat (Ma et al., 2019). This difference may arise from competition between Sb(V) and P(V) for shared uptake pathways, which limits Sb accumulation (Tisarum et al., 2015). This dose-dependent competition highlights the complexity of Sb(V) uptake, which is influenced by both specific transport proteins and interactions with uptake pathways of other nutrients. Additionally, Sb uptake is regulated by the plant’s energy metabolism. For example, malonic acid (C_3_H_4_O_4_) inhibits key enzymes such as succinate dehydrogenase (SDH), reducing ATP production and thereby limiting Sb(V) uptake. This indicates that Sb(V) uptake is affected not only by external competitors but also by the plant’s internal metabolic processes (Bentley, 1952).

In summary, Sb uptake is a complex and dose-dependent process influenced by competitive inhibitors, nutrient interactions, energy metabolism, and specific transport proteins. Future research should investigate these mechanisms further, especially Sb uptake and accumulation under varying environmental conditions, to inform more effective strategies for Sb remediation and phytoremediation.

#### 3.2.2 Mechanism of Sb transport

##### 3.2.2.1 Concentration-dependent translocation patterns

Sb transport in plants is influenced by concentration, chemical speciation, and physiological characteristics. The transport behavior of Sb varies significantly with its concentration. At low Sb concentrations (1–100 mg·L^-^¹), plants show a relatively high transport factor (TF ≈ 0.12), indicating efficient translocation from roots to shoots (Ma et al., 2019). This efficiency may be attributed to changes in osmotic pressure within the vascular system (xylem and phloem), which facilitate Sb transport (Haider et al., 2024). Under these conditions, Sb predominantly exists as Sb(III), with notable accumulation in stems and leaves, such as in wheat. This pattern likely results from the reduction of Sb(V) to the less toxic Sb(III) through internal redox reactions, which facilitates its translocation to aerial parts as a detoxification strategy. The predominance of Sb(III) at low concentrations suggests that plants may preferentially absorb and translocate this species to mitigate toxicity.

In contrast, at high Sb concentrations (≥200 mg·L^-^¹), the transport factor decreases significantly (TF < 0.06), indicating limited translocation and increased Sb retention in roots (Ma et al., 2019). This suggests a protective strategy by plants to limit systemic exposure to toxic Sb levels. At elevated concentrations, Sb predominantly exists as Sb(V), which has intrinsically lower mobility. Plants may oxidize the more mobile Sb(III) to Sb(V) to reduce its phytotoxic effects (Ma et al., 2019).

##### 3.2.2.2 Speciation transformation and detoxification mechanisms

The chemical speciation of Sb plays a critical role in its transport. Sb(III) is generally more readily translocated than Sb(V) (Ren et al., 2014). For instance, ryegrass roots accumulated 100 times more Sb under Sb(III) exposure than under Sb(V). Notably, under Sb(III) exposure, about 60% of Sb in roots and stems existed as Sb(III)-thiolate complexes (Haider et al., 2024; Ji et al., 2017), indicating active detoxification through reduction and complexation, which explains the high root accumulation and limited translocation.

Under high Sb(V) exposure, plants frequently reduce Sb(V) to Sb(III) and sequester it in vacuoles, thereby isolating the toxin. Studies have confirmed the presence of both Sb(III) and Sb(V) in stems and leaves of Sb(V)-treated plants, particularly in rice seedlings, suggesting that reduction occurs within these tissues (Cai et al., 2016). Vacuolar sequestration serves as a major detoxification strategy by reducing toxicity and preventing excessive accumulation.

Despite progress in understanding Sb transport, many aspects remain unclear. In particular, the molecular mechanisms—including key transport proteins involved in root uptake and shoot translocation—are still largely unknown. Although redox transformations of Sb are relatively well understood, interspecies differences warrant further investigation. Future research should focus on the transport and metabolic pathways of various Sb species, particularly how plants adapt to high Sb levels and minimize its accumulation. Additionally, investigating vacuolar storage mechanisms may offer new insights for mitigating heavy metal contamination.

### 3.3 As uptake and transport mechanisms

#### 3.3.1 Mechanisms of As uptake

**Figure 3:**
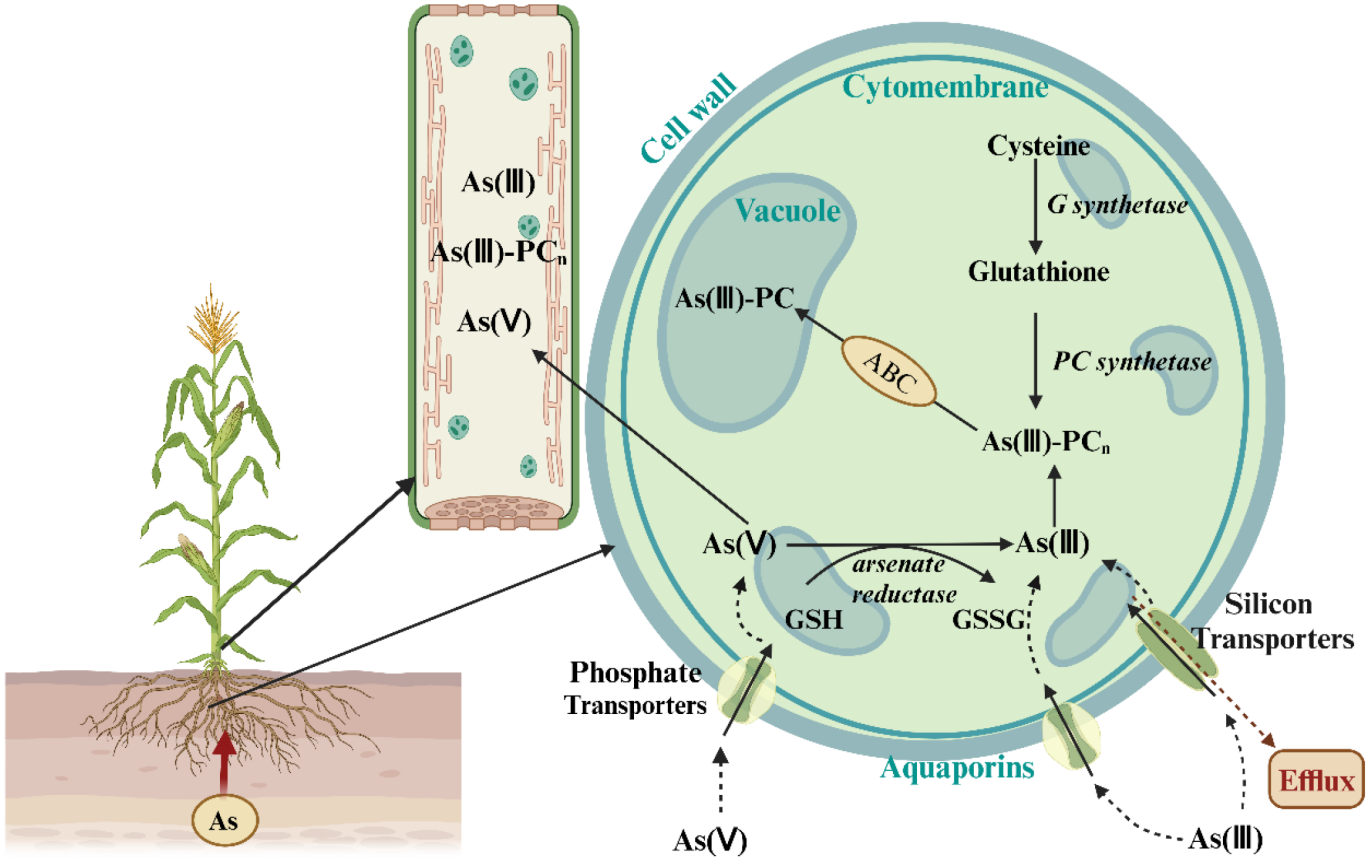
The As(III) and As(V) uptake pathways in crops, illustrating the transport from roots to stems. The diagram shows As(III) uptake predominantly through aquaporins and As(V) uptake primarily through phosphate transporters. The figure also indicates the transformation and redistribution of As species within the plant. Adapted from Allevato et al. (2019).

Arsenic is taken up by crops primarily in two forms: As(V) and As(III). As(V) is the predominant form of arsenic and structurally and chemically resembles inorganic phosphate (Pi). It primarily enters plant roots via phosphate transporters (Pht), competing with Pi for the same transport carriers in the root plasma membrane (Kumar et al., 2022). Experiments with rice mutants have shown that knocking out the phosphate transporter OsPht1;8 blocks the uptake of both Pi and As(V), thereby increasing rice tolerance to As(V) (Wang et al., 2016). Under hypoxic conditions, such as those in rice paddies, As(III) uptake becomes dominant. In this case, As(III) enters plant roots in its neutral form, H_3_AsO_3_, through the glycerol transport channel in the root plasma membrane. This process is mediated mainly by the nodulin-like intrinsic protein 26 (NIP26) family (Fox et al., 2017; Katsuhara et al., 2014; Mukhopadhyay et al., 2014; Srivastava et al., 2013). Plasma membrane intrinsic proteins (PIP) and vacuolar membrane intrinsic proteins (TIP) also participate in As(III) uptake. In rice, the low silicon 1 (OsLsi1) transporter (also known as OsNIP2;1) is the main pathway for As(III) uptake under hypoxic conditions (Bakhat et al., 2017). Methylated arsenic species (e.g., MMA and DMA) can also enter rice roots via water channel proteins, such as OsLsi1 (Li et al., 2009) (**Figure 3**). Crops absorb inorganic arsenic at a significantly higher rate than organic arsenic (Raab et al., 2007).

In summary, As uptake by crops is influenced by both its chemical form and environmental conditions. As(V) is taken up mainly through phosphate transporters, while under hypoxic conditions, As(III) is taken up via glycerol transport channels and other synergistic proteins. Understanding As uptake mechanisms provides a theoretical basis for improving crop tolerance to arsenic contamination and for better management practices.

#### 3.3.2 Mechanisms of As transport

In most plants, As is primarily transported in the xylem as As(III), with its proportion in xylem sap ranging from 60% to 100% (Li et al., 2016). In hyperaccumulators like *Pteris vittata* (Chinese brake fern), over 80% of taken up As is transported to the aboveground parts. In non-accumulators, such as Arabidopsis and rice, only 5-10% of As is transported to the leaves (Kumar et al., 2022). This highlights the stronger As transport capability of hyperaccumulators, likely due to specific transport proteins and mechanisms (Ali et al., 2009). Some aquaporins, such as NIP2;1 and NIP3;1, not only participate in As uptake but also play a key role in long-distance transport from roots to aboveground parts (Ma et al., 2008).

In addition to its long-distance transport through the xylem, arsenic (As) is also redistributed within plants via the phloem, especially during the reproductive stage when it is directed toward seeds. Similar to the transport of minerals, sugars, and amino acids, As is redistributed through the phloem from source tissues (e.g., leaves) to sink tissues (e.g., seeds) have shown that the phloem-mediated transport of As(III) from flag leaves to rice grains accounts for approximately 90% of total As transport, while dimethylarsinic acid (DMA) contributes about 55%. Additionally, OsABCC1, a vacuolar arsenic transporter located in the phloem companion cells of rice, limits As translocation to seeds (Li et al., 2016). TaPHT1;9, a phosphate transporter in wheat, has been identified as a key protein responsible for As(V) uptake. As(V) is primarily absorbed through phosphate transporters such as TaPHT1;9, which exhibits a higher affinity for As(V) than other homologs. This is attributed to the structural similarity between As(V) and phosphate ions (PO_4_³^-^), which enables shared transport pathways. Yeast mutant assays and BSMV-VIGS experiments demonstrate that TaPHT1;9 plays a crucial role in As(V) uptake and enhances plant tolerance to arsenic. TaPHT1;9 contributes to arsenic tolerance by regulating As(V) uptake, minimizing its accumulation in sensitive tissues such as roots and leaves, and promoting a more favorable distribution throughout the plant. In wheat mutants lacking TaPHT1;9, As uptake is significantly reduced, whereas overexpression of TaPHT1;9 in rice results in increased As accumulation. These findings confirm that TaPHT1;9 is essential for As(V) uptake and plays a vital role in enhancing plant tolerance to arsenic contamination (Wang et al., 2023b).

### 3.4 Uptake and transport of Sb and As in crops under co-contamination

Under Sb-As co-contamination, the coexistence of these elements can alter the plant’s uptake of both Sb and As, with high concentrations significantly increasing their toxic effects. Crop uptake of these elements typically shows either synergistic or antagonistic interactions. In terms of synergistic effects, As bioavailability is higher under co-contamination, and co-adsorption of As and Sb in the root system may enhance As uptake efficiency. Despite high Sb concentrations in soil, As accumulation in rice roots and grains is significantly higher than Sb (Wu et al., 2019). Additionally, As(V) can increase Sb uptake by altering the cell membrane’s integrity and permeability, a phenomenon especially evident in hyperaccumulating ferns (Mirza et al., 2017b; Müller et al., 2013). For instance, As promotes Sb uptake and accumulation in *Pteris vittata*, while Sb presence also enhances As uptake by the plant. This process is influenced by synergistic morphological transformations: *Pteris vittata* strongly reduces As(V) in roots, and As(III) presence may decrease Sb(V) adsorption through chemical competition, enhancing Sb bioavailability (Wan et al., 2016). Wan et al. (2017) confirmed that As reduction enhances Sb transport efficiency in plants, reflecting synergistic uptake of both elements under co-contamination conditions.

Antagonistic effects are primarily observed in several ways. First, differences in uptake and transport mechanisms between Sb and As lead to antagonistic interactions. For example, in *Pteris vittata*, Sb and As uptake and transport mechanisms differ significantly. *Pteris vittata* absorbs As(III) and transports it to above-ground parts, while Sb primarily exists as Sb(V), with lower transport efficiency, leading to most Sb being retained in roots (Wan et al., 2016). Ji et al. (2017) indicate that these transport mechanism differences limit Sb transport, exhibiting an antagonistic relationship between Sb and As. Moreover, due to the similar chemical forms of As and Sb in soil, competition occurs in plant uptake mechanisms. For example, As(V) is taken up mainly through phosphate transporters, while Sb(V) may enter the plant root system via the same pathway (Feng et al., 2019; Kumar et al., 2022). The antagonistic effect is also evident in the conversion efficiency between different forms. As is more readily converted to As(III) within the plant and transported upwards, while Sb is less easily reduced to Sb(III) and mainly remains as Sb(V) in roots. This difference in transformation efficiency hinders Sb upward transport, leading to antagonistic uptake and accumulation of As and Sb under co-contamination (Wan et al., 2016).

## 4 Toxic effect of combined Sb and As contamination on crops

Sb-As co-contamination negatively impacts crop growth and physiological functions. The coexistence of Sb and As not only inhibits plant growth and biomass but also impairs photosynthesis, affecting nutrient uptake and distribution in plants. Additionally, Sb-As co-contamination may cause synergistic toxicity, especially in As-hyperaccumulating plants, further enhancing Sb uptake. Although studies indicate that synergistic effects exacerbate plant toxicity, the specific physiological and biochemical mechanisms remain unclear and require further investigation.

### 4.1 Effects of single Sb and As contamination on crop growth

Both Sb and As in the soil significantly affect plant growth, development, and physiological functions. When Sb concentrations exceed 150 mg·kg^-^¹ in soil, it inhibits plant germination, growth, development, and photosynthesis. Sb is taken up through the roots, inducing reactive oxygen species (ROS) production, leading to cell membrane damage, disruption of chloroplast structure, and inhibition of protein synthesis and nutrient uptake, ultimately reducing plant biomass and yield (Cai et al., 2016; Feng et al., 2020, Feng et al., 2013b; Zhou et al., 2018). For example, Pan et al. (2011) found that at 1000 mg·kg^-^¹ Sb concentrations in soil, the growth and biomass of maize seedlings were significantly reduced. Furthermore, different crops exhibit varying levels of tolerance to Sb. For instance, root growth inhibition in rapeseed under Sb stress is more pronounced than in radish (Liang et al., 2018), while crops like sunflower and maize show more tolerance at lower Sb concentrations (Tschan et al., 2010; Vaculík et al., 2015).

Sb toxicity sensitivity varies across plant parts. For example, Sb toxicity is greater in the roots than in the stems of mung bean, Chinese cabbage, cucumber, and wheat (Baek et al., 2014). When Sb concentration exceeds 30 μmol·L^-^¹, leaf growth of Ficus tikoua is significantly inhibited, while roots and stems show no changes (Chai et al., 2017). In antimony mine tailings, *Achillea ageratum L*. accumulates 367 mg·kg^-^¹ Sb in its basal leaves, while Sb accumulation in the flower heads reaches 1105 mg·kg^-^¹ under soil Sb concentrations >9000 mg·kg^-^¹, with an extractable Sb concentration of 793 mg·kg^-^¹ (Baroni et al., 2000). This illustrates that plant parts vary in their sensitivity to Sb toxicity.

Sb stress also disrupts the uptake and distribution of essential nutrients in plants. For example, Sb contamination has been shown to reduce the uptake of essential nutrients such as calcium (Ca), potassium (K), zinc (Zn), and iron (Fe) in crops including wheat, rice, and leafy vegetables (Feng et al., 2013a; Tang et al., 2022; Zhu et al., 2020). This effect is primarily attributed to the chemical similarity between Sb and phosphate (PO_4_³^-^) and silicate (SiO_4_²^-^) ions, which compete for transporter proteins and thereby interfere with nutrient uptake. Specifically, due to its structural resemblance to PO_4_³^-^ and SiO_4_²^-^, Sb competes with essential nutrients for binding sites on transporter proteins. As a consequence, Sb disrupts the normal uptake and translocation of nutrients such as Ca, K, Zn, and Fe, ultimately impairing nutrient homeostasis in plants (Tang et al., 2023; Xiao et al., 2015). In addition, Sb contamination damages plant physiological structures, primarily by inducing stomatal closure, limiting CO_2_ uptake, and reducing photosynthetic efficiency (Vaculík et al., 2015; Zhou et al., 2018). Sb may also impair the plant vascular system, thereby hindering the transport of water and mineral nutrients (Baruah et al., 2021).

Similarly, the root system is the main site of As uptake, and As accumulation often inhibits root growth, reduces biomass, and affects plant fertility. As also affects plant growth by inhibiting cell expansion, reducing photosynthetic rate, and interfering with nutrient uptake (Biswas et al., 2015; Garg and Singla, 2011). For example, As stress inhibits root, stem, and leaf growth in crops like chickpeas and rice, significantly reducing dry and fresh weights (Mishra et al., 2017). As also alters plant metabolism, inhibits antioxidant enzyme activity, generates reactive oxygen species, and accelerates senescence and death (Finnegan and Chen, 2012). Its toxic effects also manifest as interference with water and nutrient uptake and distribution, particularly phosphate metabolism. Moreover, high As concentrations not only affect plant growth but also obstruct photosynthesis and damage chloroplast membranes (Faizan et al., 2022).

### 4.2 Effect of Sb-As co-contamination on crop growth

Sb-As co-contamination is more complex than single-element contamination due to their interactions in soil and plants, which can lead to increased, synergistic, or antagonistic toxic effects. Even at lower concentrations, co-contamination can exhibit higher toxicity. For example, the coexistence of Sb and As affects their bioavailability through competitive adsorption, which is crucial for uptake and accumulation in plants (Chang et al., 2022). Moreover, Sb-As co-contamination exacerbates plant toxicity, especially in aquatic plants like giant reed (*Arundo donax L.*) and water spinach (*Ipomoea aquatica*), where Sb and As coexistence significantly reduces dry weight and stem length (Egodawatta et al., 2018; Mirza et al., 2017a; Shetty et al., 2021).

Under co-contamination, the synergistic effects of Sb and As may enhance Sb uptake, accumulation, and transport in plants. For example, in As hyperaccumulating plants like *Pteris cretica* and *P. vittata*, As presence promotes Sb uptake (Feng et al., 2011; Müller et al., 2013), suggesting a synergistic effect that increases Sb bioavailability and toxicity. As noted by Müller et al. (2013), As(V) alters the integrity and permeability of the cell membrane, increasing Sb uptake. While As presence may increase Sb uptake, under co-contamination, the amount of As taken up by plants exceeds that of Sb. This is likely due to differences in Sb and As bioavailability in soil and plant uptake mechanisms (Ngo et al., 2016).

Co-contamination also significantly impacts nutrient uptake in plants. Under combined conditions of 5 mg·L^-^¹ Sb and 5 mg·L^-^¹ As, nutrient levels of P, K, Ca, Mg, S, and Fe in giant reed were significantly lower than under single-element contamination. This may be due to the stronger stress response induced by co-contamination, which affects nutrient uptake and distribution (Shetty et al., 2021). Long-term exposure to co-contamination may lead to root tip lignification in plants, which, while aiding in defense against heavy metal toxicity, may hinder nutrient uptake (Shetty et al., 2021).

Although studies show that Sb-As co-contamination significantly affects crop growth, the specific physiological and biochemical mechanisms require further investigation. Current literature mainly focuses on changes in uptake and toxicity but lacks systematic studies on the underlying mechanisms. Future research should focus on the specific mechanisms of co-contamination on plant growth and deepen our understanding of Sb and As interactions and their impact on crop growth.

## 5 Conclusion and prospects

Sb and As, chemically similar elements in the same group, pose a significant threat to soil-crop system safety and agricultural product health due to co-contamination. This paper systematically reviews the sources, morphological transformations, and bioavailability regulation of Sb and As in soil-crop systems, highlighting the following key conclusions:

1. Uptake pathway differences: As(V) is taken up by plants via phosphate transporters (e.g., OsPht1;8), while the transport mechanism of Sb(V) is not fully understood and may be independent of the phosphate system, indicating a potential unique pathway for Sb.
2. Interactive effects of co-contamination: As enhances Sb accumulation by altering cell membrane permeability (e.g., in hyperaccumulators like *Pteris vittata*). However, differences in morphological transformation efficiencies (As easily reduces to As(III) and moves upward, while Sb remains in the root as Sb(V)) lead to antagonistic effects, influencing pollutant accumulation in crops.
3. **Complexity of toxicity responses**: Under co-contamination, Sb and As synergistically exacerbate oxidative damage (e.g., ROS bursts and photosynthetic inhibition), with soil physicochemical properties (e.g., pH, Eh) and crop types (e.g., rice vs leafy vegetables) significantly influencing toxicity thresholds.

Given existing research gaps, future efforts should focus on the following directions to address the challenges of Sb-As co-contamination:

(1) In-depth study of Sb and As uptake and transport mechanisms: Although As uptake mechanisms are well studied, the uptake pathways of Sb and its transport within plants remain unclear. Under co-contamination, Sb and As may influence each other’s accumulation and distribution through competitive or synergistic uptake. Future studies should employ competitive inhibition experiments and transporter screening to clarify whether Sb(V) hijacks other ion channels (e.g., sulfate transporters) or relies on novel systems. Quantifying Sb-As competitive adsorption at the rhizosphere interface will reveal real-time changes in their bioavailability under co-contamination.
(2) Exploration of synergistic toxicity mechanisms: Sb-As co-contamination may intensify oxidative stress, inhibit photosynthesis, and disrupt nutrient uptake, severely damaging plant growth. Future research should systematically uncover the interaction mechanisms of these elements in plants, clarifying how co-contamination exacerbates toxicity by enhancing ROS generation, damaging photosynthesis, and disrupting metabolic pathways.
(3) Optimization of soil remediation techniques: Current methods, such as phytoremediation and chemical fixation, primarily target single pollutants and have limited efficacy in co-contamination environments. Future research should explore multi-faceted strategies, integrating phytoremediation, microbial remediation, and chemical methods to optimize remediation technologies in soils with co-contamination. Additionally, the efficiency of different methods in removing Sb and As from soil should be evaluated, along with strategies to reduce their bioavailability in plants.
(4) Establishment of a comprehensive environmental monitoring and risk assessment system: A comprehensive monitoring system should be established to regularly assess heavy metal levels in soil, crops, and groundwater, and set relevant safety thresholds. Specifically, for agricultural land, strengthening the monitoring of heavy metal content in agricultural products is essential for ensuring food safety. Additionally, risk assessment models for co-contamination should be developed to evaluate the potential threats of soil heavy metal contamination to ecosystems and food security.
